# Continuous recirculation of hydroponic-nutrient solutions shifts bacterial communities and induces plant-defense gene expression in lettuce

**DOI:** 10.1128/aem.01647-25

**Published:** 2026-03-13

**Authors:** Cora M. Kenderdine, Rosa E. Raudales

**Affiliations:** 1 Department of Plant Science and Landscape Architecture, University of Connecticut 7712, Storrs, Connecticut, USA; The University of Tennessee Knoxville, Knoxville, Tennessee, USA

**Keywords:** reused nutrient solution, lettuce, root rot, *Pythium*, hydroponic

## Abstract

Biotic and abiotic factors influence bacterial communities in the rhizosphere. This project aimed to characterize bacterial communities in the lettuce rhizosphere, assess the relationships between bacteria and root rot, and evaluate the impact of reused nutrient solutions on bacterial communities. Lettuce (*Lactuca sativa* cv. Rex) was grown in deep-water culture hydroponics in reused autoclaved or non-autoclaved nutrient solutions for five cycles, with or without *Pythium myriotylum*. Bacterial composition was determined via high-throughput sequencing of the 16S rRNA gene. Quantitative real-time PCR was used to detect *P. myriotylum*. Spearman’s correlation coefficient (*ρ*) was performed to assess relationships between variables. The expression of *PR1*, *PDF1.2*, and *LOX1* was quantified to determine if there were differences in plant defenses. The predominant phyla and genera across all cycles in the nutrient solution and roots were Proteobacteria (67%) and Cyanobacteria (47%) and *Acinetobacter* (13%) and *Ideonella* (22%), respectively. Bacterial communities in the nutrient solution (*R*^2^= 0.35) and roots (*R*^2^= 0.24) were significantly dissimilar between cycles. Bacterial communities were significantly dissimilar by the presence of *P. myriotylum* in nutrient solution (*R*^2^= 0.02) and roots (*R*^2^= 0.10). Bacterial correlations with health variables mostly differed by the presence/absence of *P. myriotylum*. Bacterial communities or individual bacterial isolates in recirculating hydroponic solutions that activate plant defenses resulting in the suppression or prevention of Pythium root rot in lettuce should be investigated further to be utilized as viable strains or synthesized compounds for the development of commercial products.

**IMPORTANCE:**

*Pythium myriotylum* is the causal agent of root rot and wilt disease, which can cause significant damage to lettuce in hydroponic systems. Root rot can be challenging to treat with traditional methods once it develops, often resulting in the destruction of the entire crop. Reused nutrient solutions have been reported to harbor microorganisms that may affect disease suppression. Examining how bacterial communities in recycled nutrient solutions change and trigger plant-defense genes may contribute to the reduction of Pythium root rot and provide chemical-free and cost-effective alternatives for soilless cultivation systems. Future studies focusing on specific microorganisms and their bioactive compounds will be essential for advancing biological control methods in hydroponic crop systems.

## INTRODUCTION

A dominant group of bacteria (1–3) or a diverse community (4, 5) in the rhizosphere can affect plant growth or health. Some microbes protect against plant pathogens through a variety of mechanisms such as competition for nutrients, secretion of anti-microbial compounds, and induced systemic resistance (6, 7). Plant health depends on interactions among the plant host, rhizosphere microorganisms, environmental conditions, and pathogen (8). In hydroponic systems, recirculated nutrient solutions may harbor microorganisms that influence plant growth or suppress disease (9, 10). An emerging but insufficiently investigated approach involves augmenting resident microbial communities to foster antagonistic interactions that suppress pathogens such as *Pythium* spp. or to promote mutualistic relationships with the plant, thereby enhancing its defensive capabilities.

Lettuce (*Lactuca sativa*) is a major crop in controlled environment agriculture in the United States, with 66% of production in 2019 occurring in hydroponics (11). Closed-loop hydroponic solutions can be a source or dispersal mechanism of plant pathogens like *Pythium* spp. (12). Lettuce root rot and wilt, caused by *Pythium myriotylum,* has resulted in 50% to 100% yield loss in hydroponics (13–15). Once established, root rot control is difficult to control with conventional treatments and the entire crop might be lost. Although sanitizers such as chlorine or chlorine dioxide reduce *Pythium* populations, they can be phytotoxic at effective concentrations (16–18). Other treatments, such as ozone or UV radiation, can be costly or energy intensive. Biological fungicides offer a preventative alternative, but their effectiveness is not consistent and can negatively affect plant growth (19–21). A promising yet underexplored strategy is the enhancement of resident microbial communities with antagonistic interactions to suppress pathogens like *Pythium* spp. or mutualistic interactions with the plant to enhance plant defenses.

The application of microbial inoculants or organic amendments has been widely studied in soil-based systems for pathogen suppression (22, 23). In soilless systems, resident or introduced microorganisms have also demonstrated suppressive potential (24–27). For instance, disease incidence caused from *P. aphanidermatum* on cucumber plants was 50% to 69% lower when growing the plants with reused rockwool substrates compared with sterile rockwool (28). Similarly, 20-year-old recirculated nutrient solution resulted in *in vitro* inhibition of *Rhizoctonia solani* (56.7%) and *Fusarium foetens* (43.4%), respectively (10). These observations have been attributed to the microbial activity. However, it remains unclear how bacterial communities shift over multiple crop cycles when *Pythium* spp. are introduced and what role they play in plant health.

Bacterial communities in hydroponic systems have been shown to change in response to pathogens (3, 29), plant growth promoting microorganisms (30), and repeated reuse of nutrient solutions (9). In one study, the relative abundance of Pseudomonadaceae decreased when *P. aphanidermatum* was present in lettuce roots grown in hydroponic and aquaponic systems (3). Sheridan et al. (30) observed that commercial microbial inoculants altered the diversity and taxa composition in hydroponic solution of soybeans, wheat, durum, and potatoes—although the changes were crop dependent. In an ebb-and-flood system reusing nutrient solution across six irrigation cycles, Proteobacteria relative abundance increased after the first irrigation, while the relative abundance of Actinobacteria decreased (9). These findings suggest that bacterial communities are dynamic, but crop type, environment, and pathogen may also influence microbiome composition.

Plant growth promoting rhizobacteria (PGPR), including *Pseudomonas* and *Bacillus,* enhance plant growth and suppress disease by producing antibiotics or volatile organic compounds or by triggering the activation of induced systemic resistance (31–35). Burgos-Garay et al. (36) reported a *Pseudomonas* sp. isolate collected from recirculated nutrient solution inhibited *P. aphanidermatum* and *P. cryptoirregulare* growth *in vitro* by 17.2% and 12.8%, respectively. Thus, repeated reuse of nutrient solution may foster disease-suppressive microbial communities and serve as a source of novel biocontrol agents adapted to aquatic environments.

PGPR can also trigger the activation of plant defense responses. Microbe-associated molecular pattern molecules (MAMPs) are elicitors secreted by most microorganisms that activate plant defense mechanisms (37). Within a few minutes after MAMPS are recognized, signaling pathways are activated that lead to defense responses such as systemic acquired resistance response (SAR) and induced systemic resistance (ISR) (38). SAR is regulated by salicylic acid and is associated with the expression of pathogen-related protein 1 (*PR1*) gene (39). ISR is mediated by jasmonic acid (JA) and ethylene (40) and is monitored by the expression of plant defensin 1.2 (*PDF1.2*) for the ethylene-regulated division of the jasmonic acid pathway (41, 42), and *LOX1* encodes for a JA inducible lipoxygenase required for jasmonic acid biosynthesis elicitor (43). Lettuce inoculated with *Bacillus amyloliquefaciens* or *Paenibacillus alvei* showed the upregulation of *PR1* and *PDF1.2* following *Pythium* infection (44, 45), suggesting an enhanced defense response.

In this study, we examined how microbial communities in reused nutrient solution affect the incidence and severity of *Pythium myriotylum*-induced root rot on lettuce and/or inducing plant defense genes. We hypothesized that repeated cycles of solution reuse would reduce pathogen populations and/or activate host defense pathways. To test this hypothesis, we grew lettuce in reused autoclaved or non-autoclaved nutrient solution inoculated with or without *P. myriotylum* and assessed plant growth, disease severity, bacterial community, and gene expression of *PR1*, *PDF1.2*, *LOX1*.

## MATERIALS AND METHODS

### Experimental design

The experiment followed a full factorial design arranged as a complete randomized design (CRD). The factors were nutrient solution type (autoclaved or non-autoclaved), *Pythium myriotylum* inoculation (with or without), and recirculation cycle (Cycles 1 through 5). Each experimental unit consisted of one bucket containing four lettuce plants. There was one experimental unit per replicate with five replicates per treatment (*n* = 5).

### Greenhouse setup

Lettuce (*Lactuca sativa* cv. Rex) (Johnny’s Selected Seeds, ME, USA) was sown in 25.4 mm peat pellets (Jiffy, Netherlands), with one seed per pellet. The seedlings were maintained in black trays in the greenhouse for 14 days. Seedlings with two true leaves were transferred into the hydroponic system. Four seedlings were placed into each bucket. Each bucket contained 24 L of nutrient solution prepared with 5-12-26 at 33.7 mg·L^−1^ N and 15.5-0-0 at 91.3 mg·L^−1^ N (JR Peters Inc., PA, USA). Aeration was provided continuously using a 101.6 × 50.8 × 50.8 mm air stone (Vivosun, CA, USA) connected to an external aerator (General Hydroponics, CA, USA) and the nutrient solution was maintained at 30°C using a submersible heater (Hygger, Shenzhen Mago Trading Co., Ltd., China). The study was conducted in a polycarbonate greenhouse at the University of Connecticut (Storrs, CT) from May to October 2021. Environmental conditions were maintained with a heating set point of 18.3°C, a ventilation set point of 23.9°C, and 65% relative humidity with ~15 mol·m^−2^·d^−1^.

The experiment included six consecutive hydroponic crop cycles. The first cycle (Cycle 0) was used to establish recirculation conditions. Plant responses were not measured during Cycle 0; however, bacterial community structure was characterized to serve as a baseline for comparison with subsequent cycles. For Cycles 1 through 5, each new crop cycle began with the addition of 6 L of either reused autoclaved or non-autoclaved nutrient solution (carried over from the previous cycle) to 18 L of fresh nutrient solution, resulting in a total of 24 L per container. Autoclaved nutrient solutions were sterilized at 121°C for 15 min. This dilution approach was used to maintain consistency in nutrient levels between cycles and reduce variability caused by nutrient depletion or accumulation over time. All buckets were fitted with tight lids and spaced two feet apart to prevent cross-contamination. The five measured cycles were conducted continuously, with each cycle starting immediately after the previous harvest. The entire experimental run was repeated once, resulting in two independent trials.

### *Pythium myriotylum* inoculation

Mycelial mats of *Pythium myriotylum* (GenBank accession no. MT823157) were produced following the protocol in reference 46. Three mycelial mats per 500 mL of sterile DI water were pulsed in an Oster 14-speed blender on grate/beat for 20 s. Solutions with *Pythium myriotylum* at 5 × 10^4^ oospores per mL were prepared as inoculum (47). Once the lettuce plants were transplanted into the tanks, 500 mL of the inoculum was applied directly in each tank. The negative control consisted of applying 500 mL of sterile DI water.

### Harvest and measurements

Weekly measurements consisted of visual symptoms and water chemistry parameters, such as pH, electrical conductivity (EC), dissolved oxygen (DO), and temperature. The average values (± standard deviation) of nutrient solution chemistry parameters across the experiment were as follows: pH 6.3  ±  0.39, electrical conductivity (EC) = 1,818.2  ±  91.0 µS·cm⁻¹, dissolved oxygen (DO) = 7.3  ±  1.27  mg·L⁻¹, and temperature = 30.5 ± 1.44°C. Measurements at harvest (21 days after inoculation) included DNA extracted from the nutrient solution and roots, visual symptoms, root necrosis, shoot and root biomass, and relative greenness with a chlorophyll meter (SPAD 502 Plus; Spectrum Technologies, Inc., IL). Roots were collected and plated on selective media PARP (CMA amended with pimaricin, ampicillin, rifampicin, and pentachloronitrobenzene) (48) for the presence or absence of mycelial growth. Recovery of the pathogen from symptomatic plant tissue was achieved by placing 10 pieces of 1 cm triple-washed roots on PARP. Visual symptoms were recorded daily for wilting. The area under the disease progress curve (AUDPC) was calculated using the trapezoidal integration method (49) from the daily visual wilting assessments. Root necrosis was assessed by rating the roots of each plant at harvest using the following scale: 1 = No visible symptoms, 2 = A few roots with symptoms (1%–25% rotted), 3 = Majority of roots with symptoms (26%–50% rotted), 4 = All roots infected, cortex sloughed from major roots (51%–75% rotted), 5 = Majority roots, dead or missing (>76% rotted) (50, 51). Shoots were cut at the substrate line, and lettuce heads were weighed fresh and dry (dried at 21.1°C for 2 weeks).

### DNA extraction of nutrient solution and roots

Nutrient solution samples were collected in a 1 L sterile glass container at the time of harvest, except for cycle one pre-*Pythium* (01PP) which was collected at the start of the first cycle experiment before inoculation. Two-hundred milliliters to one liter of the solution depending on clogging capacity was poured into a 0.22 µm membrane (Pall Corporation, MA, USA) held by a filter cup placed in a flask attached to a vacuum pump. DNA extraction was conducted using the PowerWater DNA Isolation Kit (Qiagen, MD, USA). At harvest, 85 mg of lettuce roots was collected from each treatment. The lettuce roots were washed with sterile deionized water, blotted dry, and placed in a 2 mL centrifuge tube with a metal bead. The tubes were placed in liquid nitrogen and then put in a TissueLyzer II machine (Qiagen, MD, USA) for 2 min at a frequency of 30 1/s or until a fine powder was formed in the tube. DNA was extracted using DNeasy Plant Mini Kit (Qiagen, MD, USA). DNA quality was quantified with a NanoDrop One^C^ Spectrophotometer (Thermo Scientific, WI, USA). DNA samples from nutrient solution and lettuce roots were used for real-time quantitative polymerase chain reaction (real-time qPCR) for the detection of *P. myriotylum* DNA copies and submitted for Illumina-MiSeq sequencing to the UConn Microbial Analysis, Resources, and Services (MARS) laboratory for 16S rRNA gene.

### Real-time quantitative PCR

**Fig 1:**
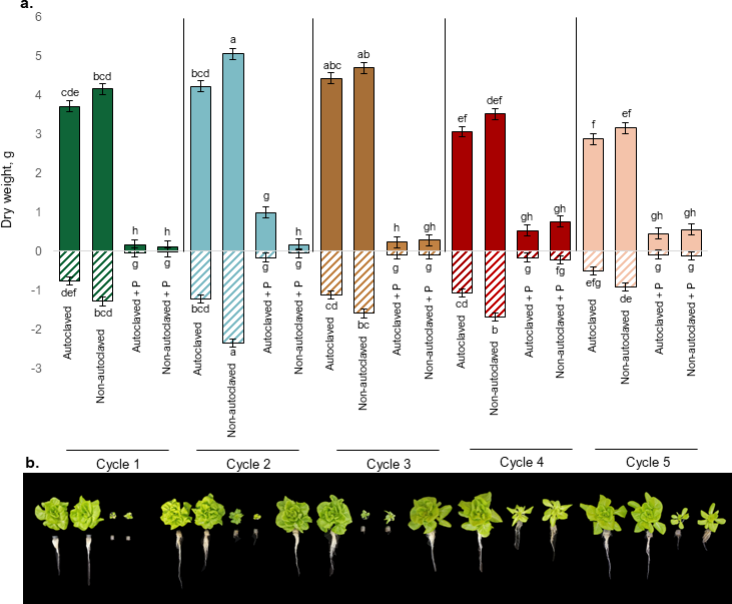
(**a**) Dry weight of lettuce shoots (above *x*-axis) and roots (below *x*-axis) in response to combinations of nutrient solution (autoclaved or non-autoclaved) *P. myriotylum* (with (+*P*) or without), and recirculation cycle (Cycles 1 through 5) in deep-water culture (*n* = 5). Data represent dry shoot weight of lettuce 35 days after sowing. Statistical comparisons reflect the interaction among all treatment combinations of nutrient solution type, *P. myriotylum* inoculation, and recirculation cycle (shoots: *P* = 0.0009, roots: *P* = 0.0355). Means with the same letters are not significantly different according to Tukey’s HSD test at *α* = 0.05 (*n* = 5). Error bars on the graph represent standard error of the mean. (**b**) Visual symptoms of lettuce inoculated with *P. myriotylum* resulted in a significant reduction in growth and wilt across all five cycles.

Real-time qPCR was conducted to quantify the amount of *P. myriotylum* DNA in different treatments for nutrient solution and root samples at harvest using species-specific primers based on internal transcribed spacer (ITS) sequence, AsPyF (5′-CTGTTCTTTCCTTGAGGTG-3′) and kkMYRR (5′-GGAGCCGAAACTCTCACAAGAC-3′) (52, 53). Quantitative PCR was performed using the CFX Connect Real-Time PCR Detection System (BioRad, CA, USA) instrument, and results were analyzed with the manufacturer’s software (CFX Manager Software). Each 10 µL reaction mixture contained 5 µL SsoAdvanced Universal SYBR Green Supermix (BioRad, CA, USA), 1 µL of 10 µM of each primer, 2 µL of genomic DNA, and 1 µL of nuclease-free water. No-template control reactions contained the same reagent concentrations, but nuclease-free water was added instead of a DNA template. Each reaction was run in technical triplicates with five biological replicates and analyzed based on absolute quantification. The thermal cycling conditions consisted of an initial denaturation step at 98°C for 3 min followed by 40 cycles each at 98°C for 15 s and 53°C for 30 s. Melt curve analysis was set at 65–95°C. Standard curves were generated by plotting the threshold cycle (Ct) of a 10-fold dilution series of known concentrations of *P. myriotylum* DNA ranging from 10 to 1 × 10^−6^ ng·µL^−1^ converted to log transformation (Fig. 1). The correlation efficiency value (*R*^2^) was 1.0, and efficiency value was 96.9%. DNA copy numbers of *P. myriotylum* were calculated by multiplying the DNA quantity (ng) by Avogadro’s constant (6.02 × 10^23^ mol^−1^) and dividing by the whole genome of *P. myriotylum* CBS 254.70 (54) in base pairs multiplied by 1 × 10^9^ and the assumed average mass of 1 bp of double stranded DNA—650 Daltons.

### Statistical analysis

Data were analyzed using SAS Version 9.4 (SAS Institute Inc., NC) to establish the effect of isolates on the response variables at (*P*) = 0.05. Homogeneity of variance and normality were checked for all measured variables using the Kolmogorov-Smirnov test, Cramer-von Mises test, and Kuiper test. Data were analyzed by analysis of variance (ANOVA), and means were separated using Tukey’s Studentized range HSD (Honestly Significant Difference) separation test using PROC MIXED. In R version 4.0.0, Kruskal-Wallis test was utilized for root necrosis data to determine differences between medians with a pairwise Dunn’s test and correcting for multiple comparisons with Holm’s method.

### Sequencing analysis of bacterial 16S rRNA gene amplicon

The Quant-iT PicoGreen kit (Invitrogen, ThermoFisher Scientific) was used to quantify DNA extracts. Partial bacterial 16S rRNA (V4) genes were amplified using 30 ng extracted DNA as template. The V4 region was amplified using 515F and 806R with Illumina adapters and dual indices (8 bp lay on 3′ [55], and 8 bp lay on the 5′ [56]). Samples were amplified in triplicate using Go-Taq DNA polymerase (Promega Corp., WI, USA) with the addition of 3.3 µg BSA (New England BioLabs, MA, USA). To overcome inhibition from host DNA, 0.1 pmol primer without the indexes or adapters was added to the mastermix. The PCR was incubated at 95°C for 3.5 min, the 30 cycles of 30 s at 95°C, 30 s at 50°C, and 90 s at 72°C, followed by final extension at 72°C for 10 min. PCR products were pooled for quantification and visualization using the QIAxcel DNA Fast Analysis (Qiagen, MD, USA). PCR products were normalized based on the concentration of DNA from 250 to 400 bp and then pooled using the Eppendorf epMotion liquid handling robot. The pooled PCR products were cleaned using Mag-Bind RXNPure Plus (Omega Bio-Tek Inc., GA, USA) according to the manufacturer’s protocol using 0.8× beads to PCR product. The cleaned pool was sequenced on the MiSeq using v2 2 × 250 bp kit (Illumina, Inc., CA, USA). Data generated from this analysis were further processed using the DADA2 pipeline and statistical software R to make graphs representing the abundance and diversity of bacterial communities in each sample.

### Sequencing data analysis

Raw sequence data for 16S rRNA reads were analyzed with the DADA2 pipeline 1.16 (57) in R version 4.0.0. An amplicon sequence variant (ASV) table was outputted from the DADA2 pipeline, which records the number of times each unique sequence variant is observed in each sample. After quality assessment, sequences were trimmed to remove low-quality reads, paired-end sequences were merged, and chimeras were removed. Chloroplast and mitochondria sequences were removed from the data set. Decontam package in R (58) identified and removed likely sequence contaminants. Read quality profiles were visualized for forward and reverse reads. Based on those visualizations, reads were filtered and trimmed with the following parameters: truncLen = (240, 160), maxEE = (2, 2), and truncQ = 2. Error rates were estimated after trimming. Filtered sequences were dereplicated, and denoising and merging were done according to the DADA2 pipeline tutorial. Chimeras were removed using the “consensus” method. All further analyses were conducted with bacterial taxa utilizing the 99% database with 7 level taxonomy using Silva version 138 (https://arb-silva.de).

### Diversity analysis

The diversity of communities was compared with beta and alpha diversity statistics and ordination plots. A permutation test (*n* = 999) was conducted using the “adonis” function in the vegan R package version 2.5–7 to test the differences between ASVs using their respective non-metric multidimensional scaling (NMDS) of unweighted UniFrac distances (59). UniFrac NMDS plots were generated with the “ordinate” function in phyloseq to indicate bacterial community composition differences across sample groups (60). Alpha diversity indices were calculated using the “estimate richness” and “plot richness” functions in the phyloseq analysis package in R version 4.0.0. Alpha diversity was measured in terms of species evenness and richness using Shannon-Wiener diversity index and Simpson’s index of diversity. Kruskal-Wallis analysis was utilized for alpha diversity indices with a pairwise Dunn’s test and correcting for multiple comparisons with Holm’s method.

### Correlations between bacterial genera with biomass and disease severity measurements

At the genus level, correlation analyses were performed for bacterial relative abundance from root samples inoculated with or without *P. myriotylum* against biomass and disease incidence and severity measurements. Spearman’s correlation coefficient was performed and visualized by R. Spearman correlation analysis made into the function “rcorr” from the package “Hmisc” (61) was used to calculate the associations. *P* values were adjusted for comparisons with the false discovery rate (FDR) algorithm after compositional transformation. The threshold to define significant corrections was the significance of the correlation adjusted *P* value <0.01. The correlation matrix of genera was visualized by the function “corrplot” (62) in R studio.

### Plant defense gene expression study setup

We conducted a separate experiment to evaluate if the changes in bacteria affected the expression of plant defense genes. The experiment was a full factorial arranged as a complete randomized design (CRD). The independent variables consisted of reused autoclaved nutrient solution and non-autoclaved nutrient solution with or without *P. myriotylum.*

Lettuce (*Lactuca sativa* cv. Rex) (Johnny’s Selected Seeds, ME, USA) seeds were immersed in 5% sodium hypochlorite (vol/vol) for 10 min and then washed with sterile DI water five times. Lettuce seeds were sown in 25.4 mm peat pellets (Jiffy, Netherlands) with one seed per peat pellet. The lettuce seedlings were maintained in black propagation trays (27.9 × 54.3 cm) in growth chambers under fluorescent and incandescent lights set at 23°C during the daytime and 18°C at night with 80% humidity June through October 2021 in Storrs, CT. After 21 days, the lettuce seedlings were transplanted into a 5 L deep-water culture (DWC) system. Each bucket had a 38 × 56 × 18 mm air stone (Pawfly, USA) hooked up to an aerator (General Hydroponics, CA, USA) with an output for each container. Each DWC bucket had 4 L of nutrient solution containing 5-12-26 at 33.7 mg.L^−1^ N and 15.5-0-0 at 91.3 mg.L^−1^ N (JR Peters Inc., PA). One liter of reused autoclaved nutrient solution and non-autoclaved nutrient solution was added to the tank depending on treatment. Three lettuce plants in a bucket constituted an experimental unit (*n* = 5). Once the lettuce was transplanted into DWC buckets, 100 mL of *P. myriotylum* at 5 × 10^4^ oospores per mL was applied directly in each tank labeled for the treatment following the protocol previously mentioned. The negative control consisted of applying 100 mL of sterile DI water.

### RNA extraction

Twenty-four hours post-inoculation, 100 mg of leaf tissue was collected in a 2 mL sterile microcentrifuge tube with a sterile metal bead and immediately put in liquid nitrogen. The tubes were quickly placed in a TissueLyzer II machine (Qiagen, MD, USA) for 2 min at a frequency of 30 1/s or until a fine powder was formed in the tube. The tubes were immediately placed in liquid nitrogen, and RNA extraction was conducted from the leaf tissue using NucleoSpin RNA Plant and Fungi Mini kit (Macherey-Nagel Inc., PA, USA). Macherey-Nagel Inc.’s rDNase set was used to purify each sample and remove any DNA contamination. RNA quality was quantified with a NanoDrop One^C^ Spectrophotometer (Thermo Scientific, WI, USA). Reverse transcriptase was performed with iScript Reverse Transcriptase Supermix (Bio-Rad, CA, USA) to convert the RNA into cDNA. The iScript Reverse Transcriptase Supermix was mixed with a 1:1,000 concentration of RNA and nuclease-free water. The reaction was placed in a T100 Thermocycler (Bio-Rad, CA, USA) at 25°C for 5 min, 46°C for 20 min, and 95°C for 1 min according to the iScript Reverse Transcriptase Supermix instructions. The cDNA was used for RT-qPCR to detect expression for selected plant defense genes.

### RT-qPCR to detect plant defense genes

To examine the expression of defense genes in lettuce, an RT-qPCR-based assay was conducted for three selected genes: pathogenesis-related protein 1 (*PR1*), plant defensin 1.2 (*PDF 1.2*), and lipoxygenase (*LOX1*) involved in salicylic acid (SA)-dependent or jasmonic acid and ethylene (JA/ET)-dependent signaling pathways (44). Amplification of the *PR1* gene was performed with forward primer (5′-GAGAAGGCCGATTATGATTA-3′) and reverse primer (5′-ATTATTGCATTGAACCCTTG-3′). Amplification of the *PDF1.2* gene was performed with forward primer (5′-GCCATCTTCTCTGCTTTTGAA-3′) and reverse primer (5′-ACACAAGACACTGCGACGAC-3′). Amplification of the *LOX1* gene was performed with the forward primer (5′-AAGAGCAGAAGCCACCCATA-3′) and reverse primer (5′-GTGGAAGGAACTGCGAGAAG-3′). Glyceraldehyde 3-phosphate dehydrogenase (*GAPDH*) was used as a reference gene with the forward primer (5′-AGGTAGCGATCAACGGATTC-3′) and reverse primer (5′-AGGTGGGATGCTTGTTTGAC-3′) (44). RT-qPCR was utilized with each gene in the CFX Connect Real-Time PCR Detection System instrument (BioRad, CA, USA). Each PCR contained: 5 µL SsoAdvanced Universal SYBR Green Supermix (BioRad, CA, USA), 1 µL forward primer, 1 µL reverse primer, 2 µL nuclease-free water, and 1 μL of cDNA template in a 10 µL reaction. Amplification of the genes was performed with the following program: 95°C for 3 min, 40 cycles of 95°C for 30 s, 60°C for 30 s, 60°C for 30 s (44). Melt curve analysis was set at 65–95°C. The 2–ΔΔCt method for relative quantification was employed. The quantity of interested genes was normalized to the quantity of the endogenous control gene (*GAPDH*) for each condition. Each treatment had five biological replicates, each with two technical replicates.

## RESULTS

### Biomass

The interaction among cycle, nutrient solution, and *Pythium* was significant for dry root and shoot weight (*P<* 0.05). Lettuce plants inoculated with *P. myriotylum* exhibited a 76% to 97% reduction in shoot dry weight and a 57% to 98% reduction in root dry weight compared to non-inoculated plants across all cycles (Fig. 1a). Shoot dry weight was higher in non-inoculated plants during cycles one to three than in cycles four and five. Among the inoculated treatments, no significant differences in root biomass were observed. Plants inoculated with *P. myriotylum* showed a marked reduction in growth (Fig. 1a and b). These results suggest that lettuce growth was predominantly influenced by pathogen presence. Additionally, shoot and root biomass were smaller in cycles four and five than in earlier cycles, regardless of treatment.

### Disease incidence and severity

Plants that were not inoculated with *P. myriotylum* showed no disease symptoms; therefore, this subset of samples was excluded from the disease incidence and severity data analysis. Only data from plants inoculated with *P. myriotylum* were included in this analysis. Plant wilting was observed 3 days after inoculation during each cycle. A significant interaction between cycle and solution for AUDPC and root necrosis was observed in the ANOVA (*P <* 0.05). AUDPC and root necrosis were highest during the first three cycles and decreased in cycles four and five (Table 1). Disease incidence was 22% higher during cycle one than cycle five for non-autoclaved nutrient solution. Analysis of variance for relative greenness (SPAD) values based on cycle and *P. myriotylum* were significantly different (*P <* 0.05) yet did not represent significant differences for solution. Relative greenness (SPAD) values ranged between 22 and 29 (data not shown). Our results indicate that disease severity measures were overall affected by cycle, but not by solution.

**TABLE 1:** Area under the disease progress curve (AUDPC), disease incidence (DI), and root necrosis (RN) measured in lettuce cv. Rex inoculated with *P. myriotylum*^*c*^

Cycle	Solution	AUDPC^*a*^	Di, %	RN^*b*^
1	Autoclaved	1,290a	92a	4.8ab
1	Non-autoclaved	1,203ab	84ab	4.8ab
2	Autoclaved	1,165ab	88ab	4.0bcd
2	Non-autoclaved	1,573a	82ab	4.7ab
3	Autoclaved	1,605a	90ab	4.9a
3	Non-autoclaved	1,348a	80ab	4.5abc
4	Autoclaved	840bc	90ab	3.8cd
4	Non-autoclaved	560cd	82ab	3.7d
5	Autoclaved	355d	80ab	3.9cd
5	Non-autoclaved	220d	72b	3.4d

^*a*^ Means within a column within each crop followed by the same letter are not significantly different according to Tukey’s HSD separation test (*α *= 0.05) (*n *= 5).

^*b*^ Root necrosis was rated according to the following scale: 1 = no visible symptoms, 2 = a few roots with symptoms (1%–25% rotted), 3 = majority of roots with symptoms (26%–50% rotted), 4 = all roots infected, cortex sloughed from major roots (51%–75% rotted), 5 = majority of roots dead or missing (>76% rotted) (51, 63). Means with the same letters are not significantly different according to Dunn’s multiple comparison with the Holm method at *α *= 0.05 (*n *= 5).

^*c*^ Statistical comparisons reflect the interaction among treatment combinations of nutrient solution type and recirculation cycle.

### Detection of *P. myriotylum* DNA copies with real time-qPCR

**Fig 2:**
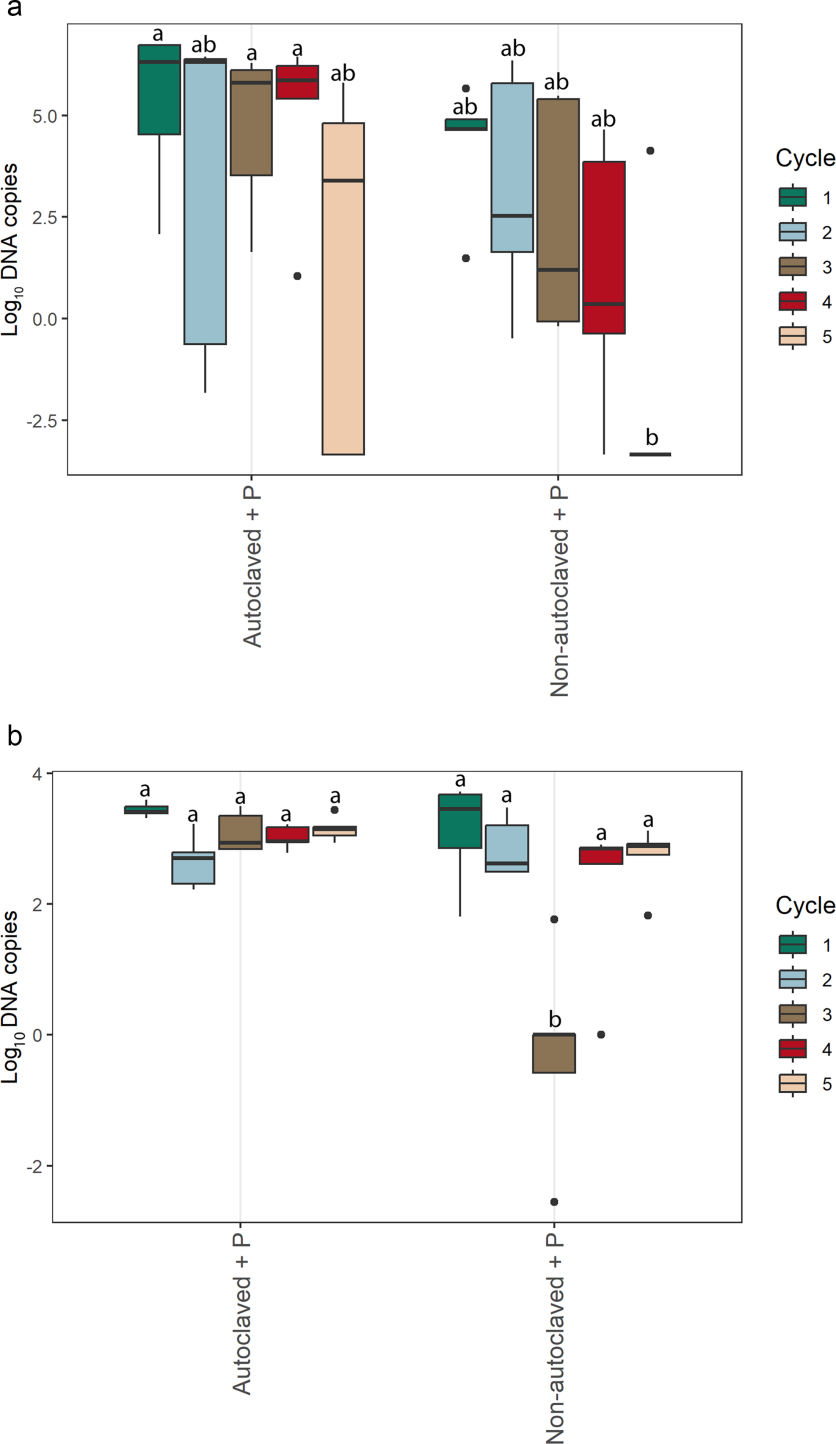
*P. myriotylum* detected in (**a**) nutrient solutions and lettuce cv. Rex (**b**) roots grown in autoclaved or non-autoclaved nutrient solution inoculated with *P. myriotylum* (+ *P*) used continuously for five growing cycles. Genomic DNA of *P. myriotylum* was quantified using real-time quantitative PCR. Means with the same letters are not significantly different according to Tukey’s HSD test at *α* = 0.05 (*n* = 5). Statistical comparisons reflect the interaction among all treatment combinations of nutrient solution type, *P. myriotylum* inoculation, and recirculation cycle (nutrient solution: *P* = 0.6137, root: *P* = <0.0001).

The efficacy (*E*) of the real time qPCR assay was 96.9%, and the correlation efficiency (*R*^2^) was 1.0 with a linear equation of *y = −*3.4*x +* 14.6. The amount of *P. myriotylum* (DNA copies) differed by solution (*P*= 0.0159) and cycle (*P*= 0.0116). *P. myriotylum* in non-autoclaved solutions with *P. myriotylum* in cycle five was significantly lower compared to autoclaved nutrient solution with *P. myriotylum* during cycle one, three, and four (Fig. 2a). In root samples from lettuce inoculated with *P. myriotylum*, there was a significant difference by solution, cycle, and the interaction of variables (*P* <0.0001). *P. myriotylum* was detected in lower amounts in roots of lettuce plants grown in non-autoclaved nutrient solution during cycle three inoculated with *P. myriotylum* compared to lettuce roots grown in all other treatments (Fig. 2b).

### Bacterial community structure

**Fig 3:**
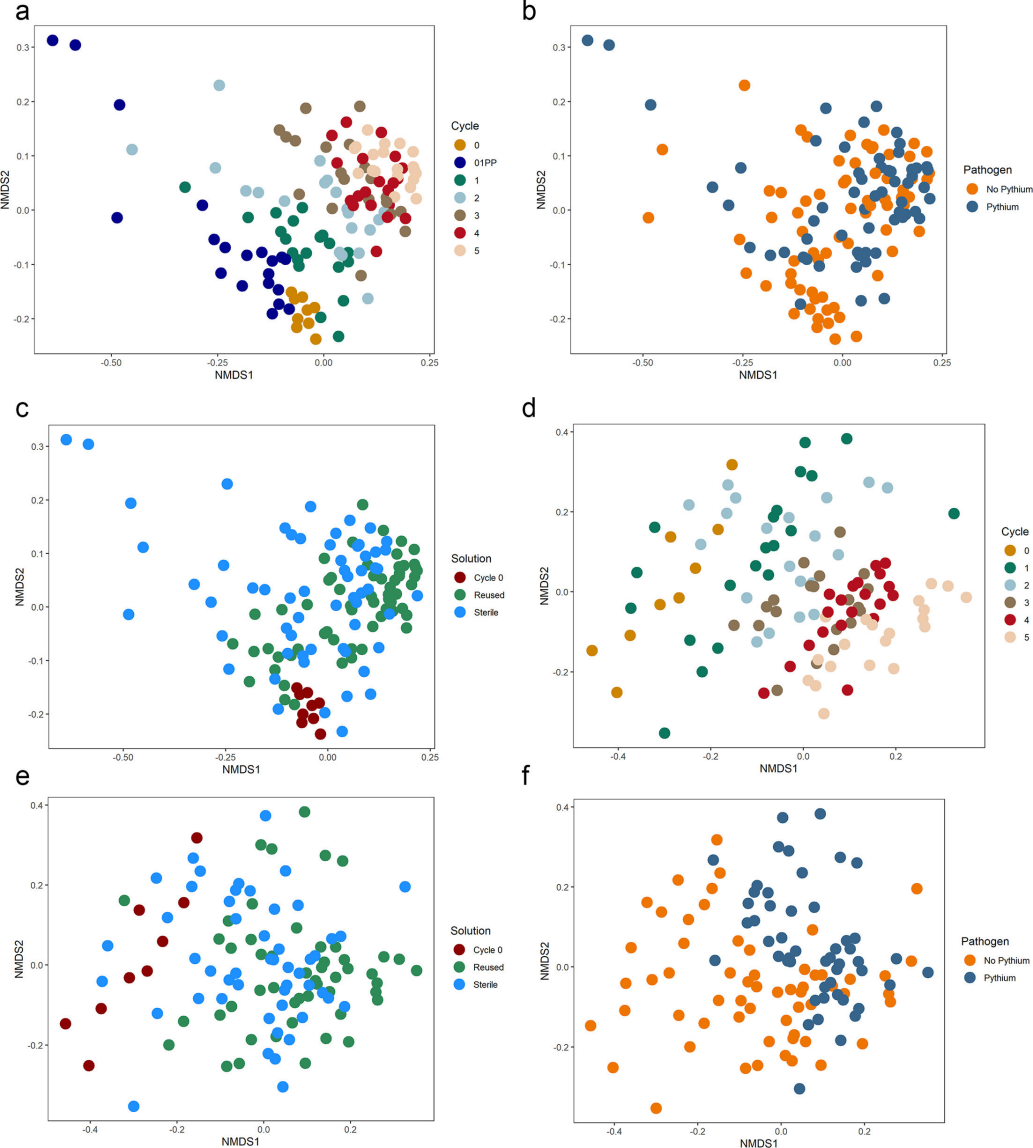
Non-metric multi-dimensional scaling (NMDS) representation of unweighted UniFrac ordination of bacterial communities separated by factors: cycle, pathogen, and solution in nutrient solution (**a–c**) and root tissue (**d–f**) across cycle, pathogen presence, and solution of lettuce grown in deep-water culture. Samples that cluster close together share a greater similarity in composition.

To characterize the impacts of cycle, solution, and pathogen presence on the lettuce rhizosphere bacterial microbiome, we analyzed beta and alpha diversity for nutrient solution and root samples. We utilized NMDS of unweighted UniFrac distance to discover the influence of sample type, cycle, solution, and pathogen on bacterial composition. We observed samples clustered primarily by sample type (nutrient solution vs roots), with beta diversity variation determined by PERMANOVA (*P*= 0.001, *R*^2^ = 0.220). The variation in beta diversity attributed to cycle and solution was greater in nutrient solution than roots, but the opposite trend was observed for pathogen (Table 2). Cycle, solution, and pathogen presence, separately, were statistically significant factors explaining beta diversity variation within both nutrient solution and roots (*P* <0.001) (Fig. 3). These results illustrate that the bacterial diversity associated with lettuce roots and the surrounding nutrient solution shifted with cycle, solution, and pathogen presence with the greatest variation attributed to cycle.

**TABLE 2:** Statistical analysis of bacterial richness and evenness with Shannon-Wiener and Simpson diversity indices among cycle, solution, and *Pythium* using Kruskal-Wallis for alpha diversity with Chi-square (*χ*2) and *P*-values, and permutational multivariate analysis of variance (PERMANOVA) of the beta-diversity based on unweighted UniFrac distances

Variable	*α* diversity				*β* diversity	
	Shannon		Simpson		PERMANOVA^*a*^	
	*χ*2	*P*	*χ*2	*P*	*R* ^2^	*P*
Nutrient solution						
Cycle	30.27	<0.0001	29.13	<0.0001	0.345	0.001
Solution	14.29	0.0008	17.82	0.0001	0.092	0.001
Cycle × Solution	39.36	<0.0001	39.94	<0.0001	0.087	0.001
*Pythium*	1.72	0.1893	1.66	0.1974	0.023	0.001
Cycle × *Pythium*	37.27	0.0002	34.63	0.0005	0.054	0.001
Solution × *Pythium*	14.79	0.0052	18.23	0.0011	0.007	0.342
Cycle × Solution × *Pythium*	68.94	<0.0001	69.29	<0.0001	0.057	0.001
Roots						
Cycle	33.47	<0.0001	41.58	<0.0001	0.242	0.001
Solution	18.88	<0.0001	21.53	<0.0001	0.081	0.001
Cycle × Solution	37.13	<0.0001	43.59	<0.0001	0.081	0.001
*Pythium*	28.44	<0.0001	22.96	<0.0001	0.101	0.001
Cycle × *Pythium*	57.47	<0.0001	60.02	<0.0001	0.062	0.002
Solution × *Pythium*	38.25	<0.0001	36.10	<0.0001	0.005	0.752
Cycle × Solution × *Pythium*	65.96	<0.0001	67.15	<0.0001	0.062	0.001

^*a*^ PERMANOVA was conducted to test for correlations between bacterial community similarity and combinations of cycle, solution, and* Pythium* factors. Permutations performed totaled 999.

**Fig 4:**
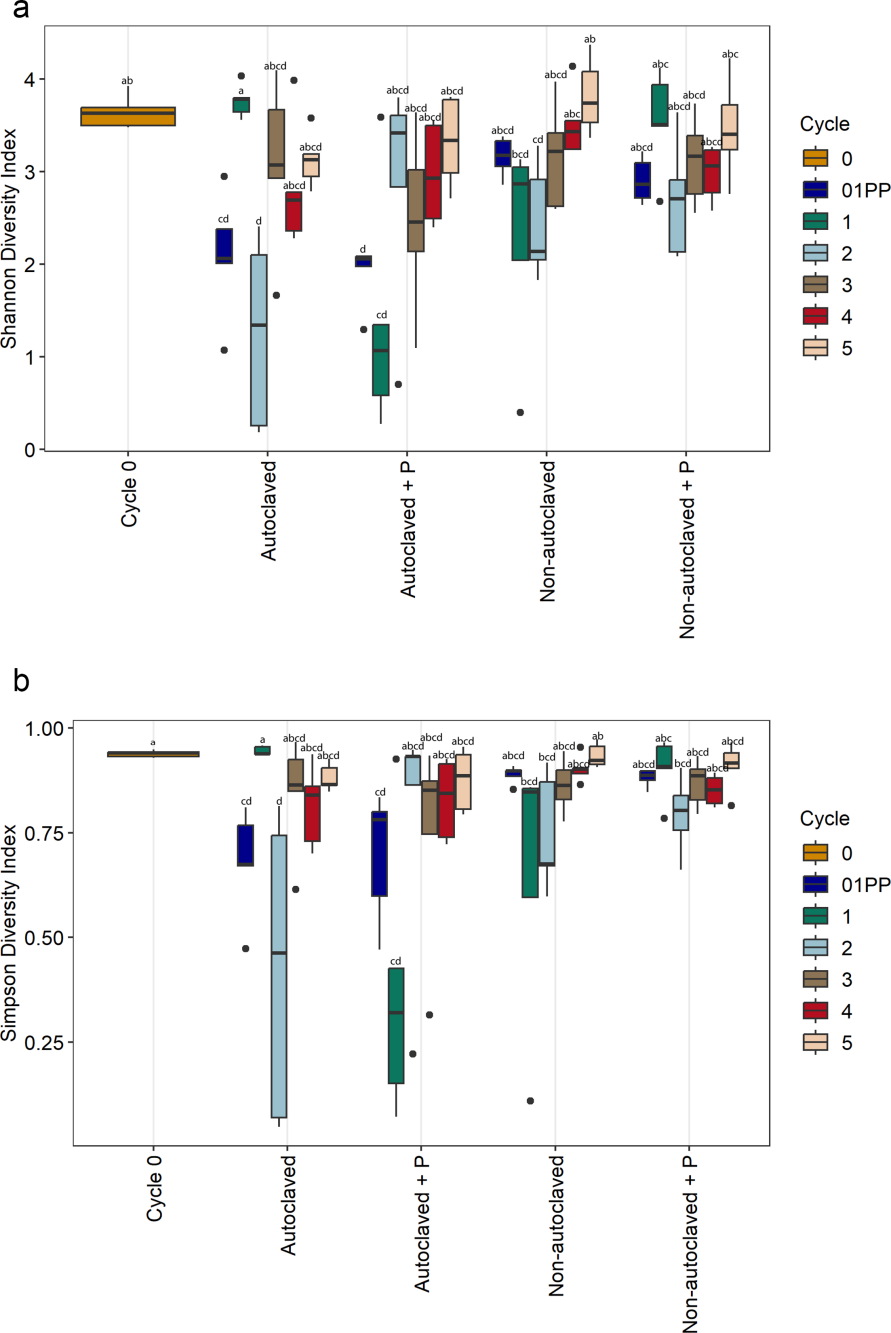
The bacterial alpha diversity metrics for nutrient solution samples based on (**a**) Shannon-Wiener and (**b**) Simpson’s diversity indices on treatments Cycle 0, non-autoclaved nutrient solution, non-autoclaved nutrient solution inoculated with *P. myriotylum*, autoclaved nutrient solution, and autoclaved nutrient solution inoculated with *P. myriotylum*. The box plots represent the observed bacterial values based on richness and evenness of different treatments. Different letters within the individual plots denote statistically significant differences between means by Kruskal–Wallis non-parametric analysis of variance followed by Dunn’s post-hoc test (*P* < 0.05) (*n* = 5).

**Fig 5:**
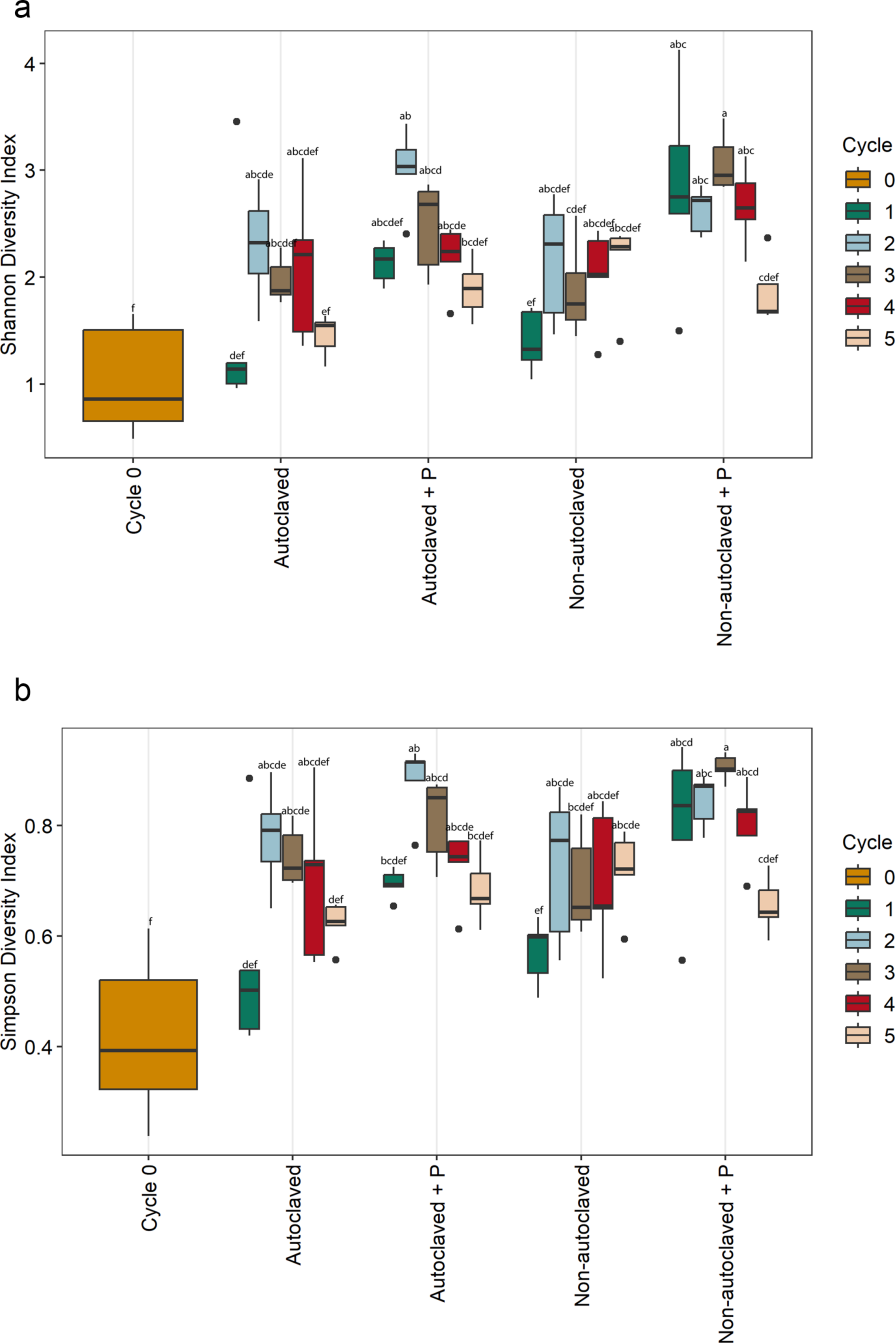
The bacterial alpha diversity metrics for root tissue samples based on (**a**) Shannon-Wiener and (**b**) Simpson’s diversity indices on treatments Cycle 0, non-autoclaved nutrient solution, non-autoclaved nutrient solution inoculated with *P. myriotylum*, autoclaved nutrient solution, and autoclaved nutrient solution inoculated with *P. myriotylum*. The box plots represent the observed bacterial values based on richness and evenness of different treatments. Different letters within the individual plots denote statistically significant differences between means by Kruskal–Wallis non-parametric analysis of variance followed by Dunn’s post-hoc test (*P* < 0.05) (*n* = 5).

Shannon and Simpson diversity indices were used to assess bacterial communities richness and evenness across factors in nutrient solution and root samples. Overall differences in alpha diversity among factors were significant for cycle and solution across sample type (Table 2). However, for pathogen presence, there was a significant effect in roots but not in nutrient solution samples. In nutrient solution, richness and evenness of bacterial communities were the same for cycle zero and non-autoclaved cycle five (Fig. 4a and b). Therefore, we conclude that recirculating nutrient solution over 21 days to complete one cycle can build up a diverse bacterial community. In root samples, there was a trend for solution with *P. myriotylum* to have higher alpha diversity than solution without pathogen presence (Fig. 5a and b), which is consistent with findings from reference 3.

### Bacterial communities via 16S rDNA sequencing

The 16S sequencing produced a total of 8,480,171 raw reads across 129 samples for nutrient solution samples. After quality filtering, 2,409 ASVs were retained. The 16S rRNA sequencing produced 3,367,687 raw reads across 109 samples for root samples. For root samples, 1,176 ASVs were retained after quality filtering.

### Bacterial composition of nutrient solution samples

**Fig 6:**
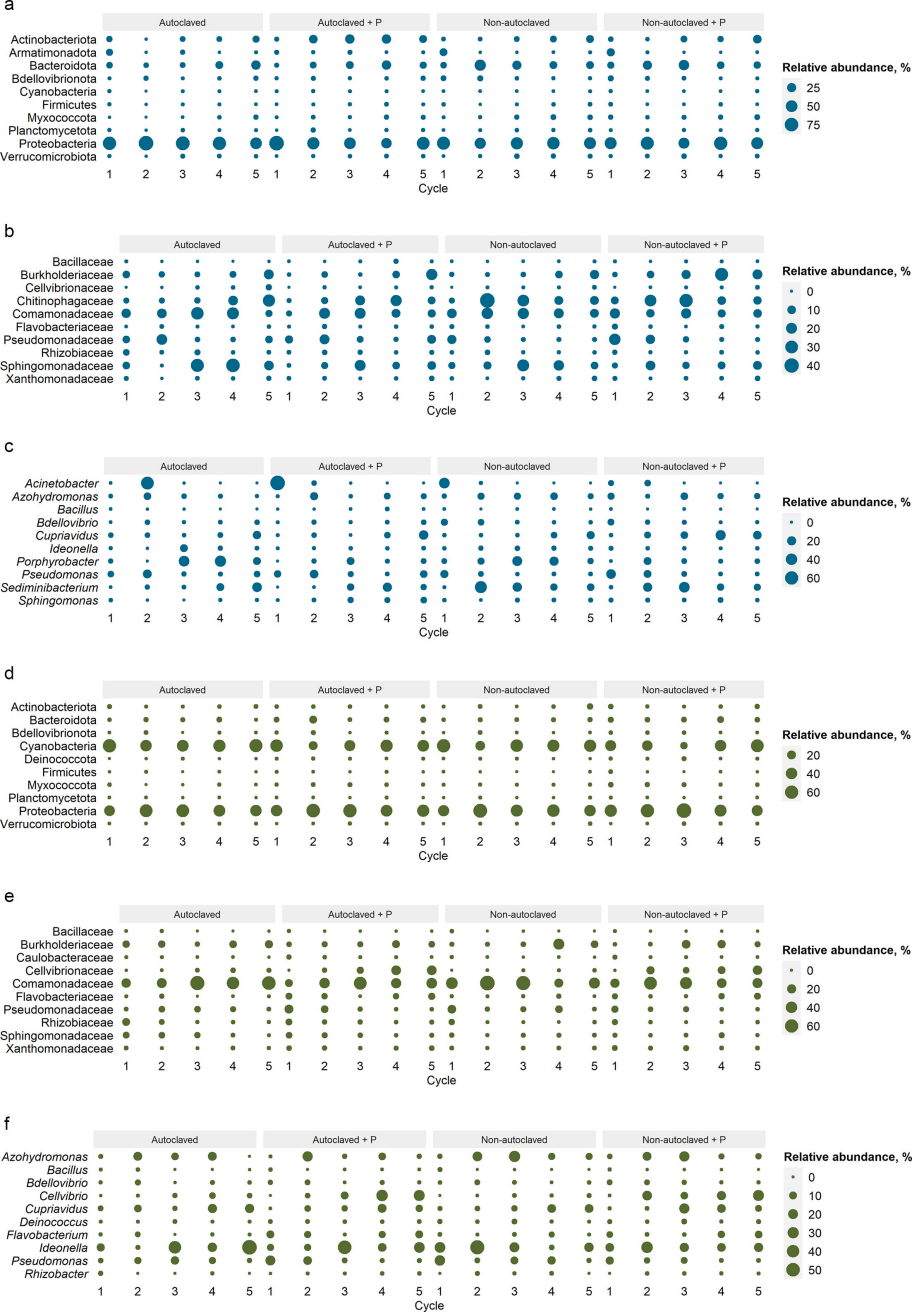
Relative abundance of the top 10 bacterial communities in nutrient solution and root tissue at the phyla level (**a, d**), family level (**b, e**), and genera level (**c, f**) detected in treatments autoclaved nutrient solution inoculated with or without *P. myriotylum* and non-autoclaved nutrient solution inoculated with or without *P. myriotylum* across five cycles.

To determine how bacterial taxa may shift in the presence or absence of *P. myriotylum* across cycles and solution, we examined the composition in nutrient solution and root samples by phyla, family, and genera. In non-autoclaved nutrient solution, Proteobacteria increased in relative abundance by 32% and 15%, while Bacteroidota and Actinobacteriota slightly decreased when *P. myriotylum* was present during cycles two and four, and a similar trend was observed in autoclaved nutrient solution during cycles one and five (Fig. 6a). The family Pseudomonadaceae increased in relative abundance in non-autoclaved nutrient solution during the first three cycles and then remained constant compared to when *P. myriotylum* was present. Yet Pseudomonadaceae decreased in relative abundance by 95% from cycle one to cycle five in non-autoclaved nutrient solution inoculated with *P. myriotylum* (Fig. 6b). The relative abundance of Comamonadaceae decreased when *P. myriotylum* was present in non-autoclaved nutrient solution for cycles two through five. Burkholderiaceae was higher in relative abundance in non-autoclaved nutrient solution when *P. myriotylum* was present across all five cycles, most notably during cycle four. The relative abundance of Rhizobiaceae in non-autoclaved nutrient solution with *P. myriotylum* was higher during cycles one, three, and four and lower for cycles two and five.

The bacterial genera *Pseudomonas* was detected in higher relative abundance in non-autoclaved and autoclaved solution inoculated with *P. myriotylum* across all cycles except for cycle four (Fig. 6c). In non-autoclaved nutrient solution, the relative abundance of bacterial genera *Porphyrobacter* was reduced from 36% to 96% when *P. myriotylum* was present compared to absent during all cycles except two. The relative abundance of *Cupriavidus* had a trend to increase when *P. myriotylum* was present in both non-autoclaved and autoclaved solution. These results demonstrate that cycle, solution, and pathogen presence relate to changes in relative bacterial community composition in nutrient solution, so next, we looked at the relative bacterial composition in root samples.

### Bacterial composition of root samples

We represented the top 10 dominant phyla, family, and genera of bacteria detected in roots grown in autoclaved or non-autoclaved nutrient solution inoculated with or without *P. myriotylum*. For roots in non-autoclaved solution with *P. myriotylum,* we observed shifts in bacterial taxa such as reduced relative abundance of Proteobacteria during cycles two, four, and five and a greater abundance of Bacteroidota across all cycles compared to when *P. myriotylum* was absent (Fig. 6d). For roots grown in both solutions inoculated with *P. myriotylum* Cellvibrionaceae was higher by 81% to 99%. Flavobacteriaceae and Xanthomonadaceae were consistently higher in relative abundance across solutions and cycles in roots with *P. myriotylum* present (Fig. 6e). Rhizobiaceae had higher relative abundance for the majority of cycles when *P. myriotylum* was present in roots grown in non-autoclaved nutrient solution and autoclaved nutrient solution. The genera *Cellvibrio* and *Flavobacterium* had higher relative abundance across solutions and all five cycles when *P. myriotylum* was present compared to when absent (Fig. 6f). *Pseudomonas* was lower in relative abundance in roots grown in autoclaved and non-autoclaved nutrient solution inoculated with *P. myriotylum* compared to solutions without the pathogen during cycles three, four, and five. *Cupriavidus* had lower relative abundance in autoclaved and non-autoclaved nutrient solution with *P. myriotylum* in all cycles except for three compared to solutions without *P. myriotylum*.

### Correlations of bacteria with biomass and disease severity measurements

**Fig 7:**
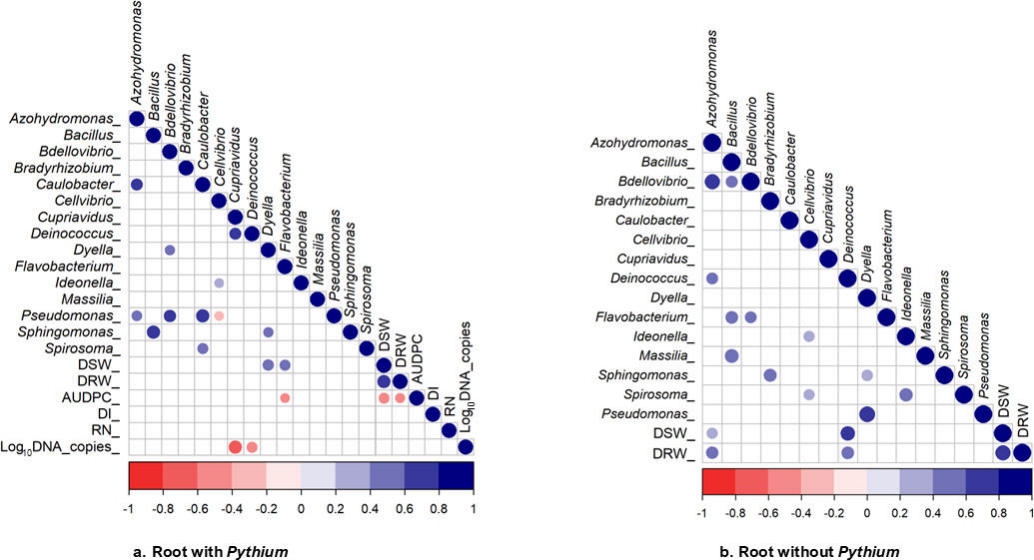
Spearman rank correlation of the dominant bacterial genera with biomass, and disease incidence and severity measures across root tissue samples with *P. myriotylum* (**a**) and without *P. myriotylum* (**b**). Blue-tinted circles represent positive correlations, and red-tinted circles represent negative correlations. Darker shades of color and larger circles represent stronger correlations.

In root samples, *P. myriotylum* affected both the diversity and relative abundance of bacterial communities. To explore potential relationships, Spearman’s correlation coefficients were calculated (Fig. 7a). In roots inoculated with *P. myriotylum*, *Pseudomonas* showed positive correlations with *Azohydromonas*, *Bdellovibrio*, and *Caulobacter* and negative correlations with *Cellvibrio*. In the absence of *P. myriotylum,* only one positive association with *Pseudomonas* was observed (Fig. 7b). While these associations highlight differences in microbial interactions depending on pathogen presence, it is important to note that correlation does not imply causation. These trends are exploratory and should be further investigated to understand their potential biological relevance.

### Expression of plant defense genes

**Fig 8:**
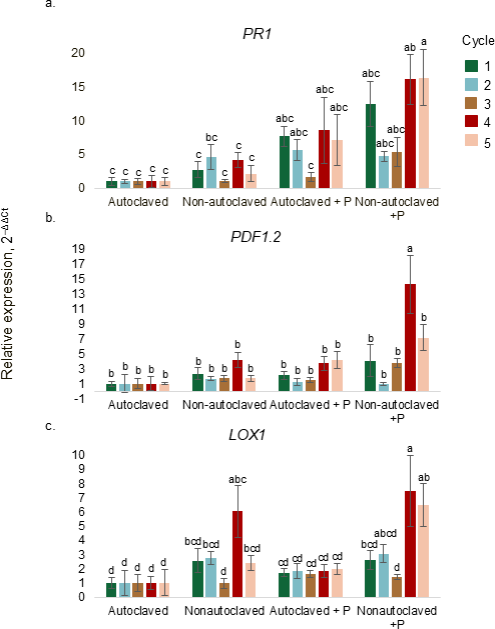
Relative expression (2^–ΔΔCt^) of plant defense genes *PR1* (**a**), *PDF1.2* (**b**), and *LOX1* (**c**) in lettuce shoots grown in deep-water culture with autoclaved or non-autoclaved nutrient solution inoculated with or without *P. myriotylum*. Autoclaved nutrient solution was the internal control. Total RNA was isolated from leaves of plants 24 h post inoculation. Transcript levels of the examined genes were normalized to the expression of *GADPH* measured in the same samples. Means with the same letters are not significantly different according to Tukey’s HSD test at *α* = 0.05 (*n* = 5). Statistical comparisons reflect the interaction among treatment combinations of nutrient solution type and recirculation cycle (*PR1: P* = 0.4312, *PDF1.2: P* = 0.1816, *LOX1: P* = 0.5371).

Lettuce plants grown in non-autoclaved solution inoculated with *P. myriotylum* displayed a higher level of *PR1* during cycle five compared with plants grown without *P. myriotylum* during all five cycles (Fig. 8a). Lettuce plants grown in non-autoclaved solution inoculated with *P. myriotylum* exhibited an upregulation of *PDF1.2* during cycle four compared to all other treatments (Fig. 8b). Lettuce grown in non-autoclaved solution inoculated with *P. myriotylum* had an upregulation of *LOX1* during cycle four compared to lettuce grown in autoclaved solution (Fig. 8c).

## DISCUSSION

In this project, we examined whether consecutive cycles of reused nutrient solution influence bacterial communities, *Pythium* root rot severity in lettuce, and the expression of plant defense genes. We observed that both bacterial community composition and disease severity were significantly influenced by the production cycle, independent of whether the nutrient solution was sterilized between cycles. Differences in microbial communities were also observed between lettuce roots infected with or without *P. myriotylum*. Notably, plant defense responses increased in *P. myriotylum*-infected plants during the fourth and fifth cycles. Reusing nutrient solutions over multiple cycles resulted in reduced disease severity and activation of plant defense genes although the exact mechanism remains unclear.

Suppressive soils have resident microbes with antagonistic modes of action against plant pathogens (64). A classic example is wheat monoculture, where disease suppression to take-all disease (*Gaeumannomyces graminis* var. *tritici*) develops over time. Disease incidence peaks during the fifth cycle and declines in subsequent cycles (65, 66). We aimed to apply this concept to soilless production by continuously growing lettuce in a closed-loop hydroponic system by reusing the nutrient solution after each crop cycle. In our study, nutrient solutions were reused through six consecutive cycles. Overtime, we observed a reduction in plant growth (Fig. 1), AUDPC, and root necrosis (Table 1). No differences in biomass or disease severity were observed between autoclaved and non-autoclaved treatments, suggesting that disease suppression may result from non-living factors in the solution. Various *Bacillus* spp., such as *B. subtilis*, produce secondary metabolites, including cyclic lipopeptides (CLPs), which have been shown to have antimicrobial properties and can activate plant defenses (67). It is possible that secondary metabolites accumulated in the reused nutrient solution over time.

We also observed increased plant defense gene expression in the later cycles in plants exposed to *P. myriotylum*. Several PGPRs, such as *Pseudomonas* and *Bacillus*, are known to induce systemic resistance in plants (68–70). In our study, *Bacillus* and *Pseudomonas* genera—known for promoting growth and disease suppression—were among the top 10 genera detected in the nutrient solution and lettuce roots. While JA–ET signaling pathways are commonly activated by PGPRs, some bacteria also induce salicylic acid (SA)-dependent defenses (71, 72). Lettuce grown in non-autoclaved nutrient solution inoculated with *P. myriotylum* exhibited increased expression of defense-related genes after cycles four and five. These bacteria may have contributed to plant defenses via activation of JA/ET pathways, as previously observed by 44, where *Bacillus amyloliquefaciens* upregulated *PDF1.2* in lettuce challenged with *Rhizoctonia solani*. Our findings suggest that microbial byproducts or plant-microbe interactions in reused nutrient solutions may contribute to disease suppression through the induction of plant defense pathways against *P. myriotylum*.

Previous studies have reported bacterial diversity and abundance of specific groups in roots differed by the presence of a pathogen (73, 74). A study looking at the endosphere of lettuce roots grown in a hydroponic and aquaponic system observed bacterial species richness and diversity was higher in plants inoculated with *Pythium aphanidermatum* vs non-inoculated plants (3). We observed a similar trend in this study, where alpha diversity in lettuce roots was slightly higher in the presence of *P. myriotylum*. These differences may result from altered root exudation in response to pathogen stress. Plants can influence rhizosphere microbial communities through the release of exudates, which may selectively recruit beneficial microbes that produce antimicrobial compounds such as coumarins and benzoxazinoids (75, 76). Future research should focus on identifying ISR-inducing bacteria and their metabolites to select candidate strains capable of reducing *Pythium* root rot.

Reusing nutrient solution did not negatively impact lettuce biomass across most cycles. This finding is relevant to growers, who may reduce water, fertilizer, and biological fungicide inputs without compromising crop performance. A slight reduction in plant biomass was observed in the last two cycles, likely due to lower temperatures (4°C ± 3°C) in the greenhouse during September and October compared with July and August. Further studies should evaluate the growth and disease suppression potential of reused nutrient solutions across different lettuce cultivars. Additional research is needed to better understand the structure and function of microbial communities in soilless systems, their interactions, and their role in plant health. 

Our study demonstrates that recirculating hydroponic nutrient solutions across multiple crop cycles can shift bacterial communities, reduce Pythium root rot severity, and enhance plant defense gene expression in lettuce. These findings suggest that microbial communities enriched through repeated nutrient solution reuse may contribute to disease suppression and offer a promising, sustainable alternative to chemical controls in soilless systems. Further research into the specific microbial taxa and metabolites involved will be key to developing effective biocontrol strategies for hydroponic crop production.

## Data Availability

Raw sequence reads were deposited in the NCBI Sequence Read Archive in BioProject PRJNA997733 with BioSample accession numbers SAMN36688063 to SAMN36688300. All computational scripts and workflow are available on GitHub (https://github.com/csmcgehee/Recirculation-hydro).
